# Biocultural Drivers of Salivary Microbiota in Australian Aboriginal and Torres Strait Islander Children

**DOI:** 10.3389/froh.2021.641328

**Published:** 2021-03-18

**Authors:** Matilda Handsley-Davis, Emily Skelly, Newell W. Johnson, Kostas Kapellas, Ratilal Lalloo, Jeroen Kroon, Laura S. Weyrich

**Affiliations:** ^1^Australian Centre for Ancient DNA, School of Biological Sciences, University of Adelaide, Adelaide, SA, Australia; ^2^Australian Research Council Centre of Excellence in Australian Biodiversity and Heritage, University of Wollongong, Wollongong, NSW, Australia; ^3^Menzies Health Institute Queensland, Griffith University, Gold Coast, QLD, Australia; ^4^School of Dentistry and Oral Health, Griffith University, Gold Coast, QLD, Australia; ^5^Faculty of Dentistry, Oral and Craniofacial Sciences, King's College London, London, United Kingdom; ^6^Indigenous Oral Health Unit, Australian Research Centre for Population Oral Health, Adelaide Dental School, University of Adelaide, Adelaide, SA, Australia; ^7^School of Dentistry, University of Queensland, Brisbane, QLD, Australia; ^8^microARCH Laboratory, Department of Anthropology and Huck Institutes of Life Sciences, The Pennsylvania State University, University Park, PA, United States

**Keywords:** bacteria, community dentistry, dental caries, ecology, microbiology

## Abstract

Australian Aboriginal and Torres Strait Islander children experience unacceptably high rates of dental caries compared to their non-Indigenous Australian counterparts. Dental caries significantly impacts the quality of life of children and their families, particularly in remote communities. While many socioeconomic and lifestyle factors impact caries risk, the central role of the oral microbiota in mediating dental caries has not been extensively investigated in these communities. Here, we examine factors that shape diversity and composition of the salivary microbiota in Aboriginal and Torres Strait Islander children and adolescents living in the remote Northern Peninsula Area (NPA) of Far North Queensland. We employed 16S ribosomal RNA amplicon sequencing to profile bacteria present in saliva collected from 205 individuals aged 4–17 years from the NPA. Higher average microbial diversity was generally linked to increased age and salivary pH, less frequent toothbrushing, and proxies for lower socioeconomic status (SES). Differences in microbial composition were significantly related to age, salivary pH, SES proxies, and active dental caries. Notably, a feature classified as *Streptococcus sobrinus* increased in abundance in children who reported less frequent tooth brushing. A specific *Veillonella* feature was associated with caries presence, while features classified as *Actinobacillus/Haemophilus* and *Leptotrichia* were associated with absence of caries; a *Lactobacillus gasseri* feature increased in abundance in severe caries. Finally, we statistically assessed the interplay between dental caries and caries risk factors in shaping the oral microbiota. These data provide a detailed understanding of biological, behavioral, and socioeconomic factors that shape the oral microbiota and may underpin caries development in this group. This information can be used in the future to improve tailored caries prevention and management options for Australian Aboriginal and Torres Strait Islander children and communities.

## Introduction

Dental caries is a highly prevalent oral disease that severely impacts children and families' quality of life [1, 2]. Australian Aboriginal and Torres Strait Islander children have higher rates of dental caries, and of untreated caries, than non-Indigenous Australian children [3]. Additionally, Aboriginal and Torres Strait Islander children in remote communities experience worse oral health than their urban counterparts [4, 5]. For example, a 2006 survey of child caries experience in a remote Aboriginal and Torres Strait Islander community in the Northern Peninsula Area (NPA) of Far North Queensland found that caries experience in NPA children was over four times the national average [6]. Caries incidence in this community remained unacceptably high as of 2015 [7]. Risk factors for caries include lifestyle (e.g., oral hygiene, diet, fluoride exposure) and underlying host susceptibility (e.g., immune factors, prevalence of caries-promoting oral bacteria) [8, 9]. Many of these factors are prevalent for Aboriginal and Torres Strait Islander children [10].

Recent research describes how caries is mediated by the oral microbiota – the community of microorganisms inhabiting the human mouth [11]. Although the factors shaping Aboriginal and Torres Strait Islander child oral microbiota have not yet been explored, changes in microbiota composition associated with caries initiation and progression have previously been reported in other groups [12–16]. Many host-related and environmental factors, including vertical transmission, diet, and antibiotic use have been reported to influence the diversity and composition of an individual's oral microbiota [17–23]. In turn, these factors may contribute to broader-scale microbiota differences among members of human groups with different heritage and lifestyles [24, 25]. For example, studies from Venezuela, the Philippines and Uganda have reported differences in saliva microbiota diversity and composition between Indigenous groups with hunter-gatherer lifestyles and counterparts living agricultural or industrialized lifestyles [26–28]. Furthermore, our previous work identified systematic differences between the oral microbiota of Indigenous Australian and non-Indigenous adults, including differences linked to oral health [29]. These findings raise the possibility that Aboriginal and Torres Strait Islander children may have unique oral microbiota signals linked to caries development.

Here, we investigate the oral microbiota in children aged 4–17 from the NPA using stimulated saliva and extensive metadata from dental examinations and participant questionnaires. Our objective was to examine whether any metadata factors were linked to salivary microbiota diversity and composition and thereby to better understand how the microbiota, lifestyle and socioeconomic factors, and oral disease interact in this population. We explored factors influencing the salivary microbiota and identified factors linked to dental caries in the absence of regular professional dental care. Our study provides important baseline data to better understand how the oral microbiota and its relationship to caries is shaped in a remote Aboriginal and Torres Strait Islander community and how the oral microbiota may be harnessed to improve Indigenous oral health. Such data will be crucial to ensure that future microbiota-aware therapies for dental caries and other oral diseases are also relevant to these communities.

## Materials and Methods

### Ethical Approval

In planning the study, extensive consultations were held with Elders and community members. Data reported in this manuscript are from a single baseline survey of oral and general health, conducted in 2015, prior to the implementation of preventive measures against dental caries. Feedback from the overall study was provided to the community in 2018 and 2019. Ethics approval was granted by the Griffith University Human Research Ethics Committee (GU Ref No: DOH/05/15/HREC); the Far North Queensland (FNQ) Human Research Ethics Committee (FNQ HREC/15QCH/39-970); the Department of Education and Training (Queensland Government) to approach participants at the schools; and the Torres and Cape Hospital and Health Service for Site Specific Approval. All surveys were conducted with the full understanding and written consent of parents/guardians of children from the three school campuses in the NPA, and with support of the Principal and teaching staff. We have worked closely with Community Health staff, and regularly consulted with the Mayor and Community over the years.

### Participant Recruitment and Data Collection

Participating children aged 4–17 were recruited and sampled at a single timepoint as the baseline for a larger clinical trial examining the impact of an annual caries preventive intervention in the NPA (ANZCTR no. ACTRN12615000693527, registered 3 July 2015) [4]. Saliva samples were collected by chewing on paraffin wax for 5 min while expectorating into a sterile cup. Total saliva volume produced was recorded and 2 mL of saliva was transferred to an OMNIgene OM-501 collection tube (DNA Genotek) and stored according to manufacturer's instructions. Samples were collected throughout school hours; due to complex field conditions, information on last food intake was not collected. Dental examinations were performed by trained and calibrated examiners as previously described [30]. For microbiota analyses, caries severity categories were assigned based on International Caries Detection and Assessment System (ICDAS) II codes [31] as follows: 0 = sound, 1–2 = incipient, 3–4 = moderate, 5–6 = severe; caries status was assigned based on ICDAS II codes as follows: 0 = caries-free, 1–6 = caries-active; categories were assigned to saliva samples based on the highest ICDAS II code recorded for that child. As compressed air was not available during examinations, teeth were dried with gauze prior to ICDAS assessment. Questionnaires detailing oral health behaviors, diet, emotional well-being, and oral health impact on quality of life were completed by participants or caregivers (Supplementary Table 3). Specifically, the validated CHU-9D [32] and OHIP-14 [33] systems were used to collect data on general child quality of life and oral health-related quality of life.

### DNA Extraction and Sequencing

A total of 255 saliva samples were used for DNA extraction and sequencing, from participants whose parents had consented to oral microbiota analysis. Genomic DNA was extracted from saliva samples in a clean facility at the University of Adelaide using the Roche High Pure PCR Template Preparation Kit (Roche Life Sciences). Two extraction blank controls (EBCs, i.e., empty tubes) were included for every 22 saliva samples. The V4 region of the bacterial 16S rRNA gene was amplified using uniquely barcoded reverse primers for each sample, as previously described [34]. No-template controls (NTCs) were processed alongside each amplification. Amplified, barcoded DNA was quantified using Qubit (ThermoFisher Scientific), pooled at equal relative concentrations, cleaned using Ampure magnetic beads (New England Biolabs), and quantified using TapeStation (Agilent). Paired-end 150 bp sequencing was performed using an Illumina MiSeq.

### Sequence Data Processing

Data were processed using QIIME2 (v. 2018.8) [35]. Briefly, sequences were demultiplexed, denoised using the *q2-deblur* plugin [36], and assigned taxonomy using a classifier trained on the SILVA 132 database, selected as the most suitable taxonomic database as it contains sequences from a wide range of sample types. Key taxonomic results were also compared against the Human Oral Microbiome Database (HOMD) v15.1 (Supplementary Tables 4, 6). Five samples were removed from further analysis due to insufficient data or withdrawal of consent. Amplicon sequence variants (referred to herein as microbial “features”) observed <3 times were removed from the dataset. Detection and removal of putative contaminant features at the 0.5 threshold (i.e., features that were more prevalent in negative controls than in samples were considered contaminants) was performed using *qiime2R* [37] and *decontam* [38].

### Microbiota Analysis

Data were analyzed using QIIME2 (v. 2019.7). Only samples with at least 30,000 sequences per sample were retained for microbiota analysis, leading to the removal of 42 saliva samples due to insufficient sequence depth; three samples from individuals who had contributed two saliva samples each were also removed, leaving a total of 205 samples. Samples with unknown or unrecorded values for a given metadata factor were removed prior to analysis, and categorical metadata factors needed at least 10 samples in each group for significance testing. Alpha diversity (within sample diversity; Faith's phylogenetic diversity [39]) and beta diversity (between sample diversity; unweighted UniFrac [40]) were calculated and statistically examined at the feature (amplicon sequence variant) level using the *q2-diversity* plugin, randomly subsampling data to 30,000 sequences per sample. These metrics were chosen because they incorporate phylogenetic as well as non-phylogenetic information about the diversity of the samples. Statistical significance was determined non-parametrically using Spearman correlation (for continuous variables) or Kruskal-Wallis (for categorical variables) [41] tests for alpha diversity and adonis tests [42, 43] for beta diversity. Metadata categories that returned a significant or near-significant result in adonis tests were further investigated using PERMANOVA [42] and permdisp tests [44]. Features with <10 observations and/or present in <5 samples were removed prior to ANCOM testing using *q2-composition ancom* to identify features that differed significantly in abundance across categorical sample groups [45]. Figures were constructed in RStudio [46] using the *qiime2R* [37] and *ggplot2* packages [47], or downloaded from QIIME2 View and edited for clarity using Inkscape 2.0.

## Results

### Sociodemographic Characteristics of Participants

A total of 205 saliva samples with corresponding metadata were used for microbiota analysis (Table 1). Although specific ethnicity data was not collected alongside individual saliva samples, within the NPA community 49.5% identified as Aboriginal Australian and Torres Strait Islander, 46.4% identified as Torres Strait Islander only, 1% identified as Aboriginal Australian only, and 3.1% identified as neither. A full list of metadata factors tested in microbiota analysis is given in Supplementary Table 1; further details on values recorded for continuous metadata variables are given in Supplementary Table 2.

**Table 1:** Sociodemographic characteristics of study participants.

**Category**	**Mean**	**Standard deviation**	**Range**
**Continuous variables**			
Age	8.53	3.53	4–17
Saliva flow rate	7.05	0.49	5.4–7.8
Saliva pH	5.77	3.00	0.5–9.5
Total carious surfaces	9.68	9.8	0–62
**Category**	**Values**	**n**	**%**
**Categorical variables**			
**Sex**			
	Female	115	56.1%
	Male	90	44.0%
**Caries status**			
	Caries-free	17	8.3%
	Caries-active	184	90%
	Unknown or not recorded	4	2.0%
**Caries severity**			
	Caries-free (ICDAS 0)	17	8.3%
	Incipient caries (ICDAS 1–2)	37	18.0%
	Moderate caries (ICDAS 3–4)	47	22.9%
	Severe caries (ICDAS 5–6)	100	48.8%
	Unknown or not recorded	4	2.0%
**Household size**			
	1–5	86	42.0%
	6–10	96	46.8%
	More than 10	10	4.9%
	Unknown or not recorded	13	6.3%
**Household employment status**			
	No people work	18	8.8%
	At least one person works	172	83.9%
	Unknown or not recorded	15	7.3%
**Soft drink consumption**			
	Yes	156	76.1%
	No	41	20.0%
	Unknown or not recorded	8	3.9%
**Daily toothbrushing**			
	Less than once	16	7.8%
	Once	34	16.6%
	Twice	130	63.4%
	More than twice	17	8.3%
	Unknown or not recorded	8	3.9%
Total		205	100%

*A summary of important sociodemographic characteristics of individuals who donated the 205 saliva samples used for microbiota analysis is presented in this table. For continuous variables, the mean, standard deviation and range values are reported, rounded to two decimal places. A detailed record of data for continuous variables is presented in Supplementary Table 2. For continuous categories, the number of samples and percentage of the total in each possible value are reported; percentage values are rounded to one decimal place and may not add to exactly 100%*.

### Background DNA Had a Limited Effect on Salivary Microbiota

Because environmental and laboratory-based contamination can significantly impact microbiota studies [48, 49], we used negative controls (EBCs and NTCs) to track contamination in our study. We verified significant differences in diversity (H = 62.67, *p* = 2.44 ×10^−15^) and composition (*R*^2^ = 0.21, *p* = 0.001) (Supplementary Figure 1A) between saliva and negative controls. We used *decontam* to statistically identify and remove 39 contaminant microbial features [38] (Supplementary Figure 1B; Supplementary Table 3).

### The Salivary Microbiota of NPA Children Is Dominated by Typical Human Oral Taxa

**Figure 1:**
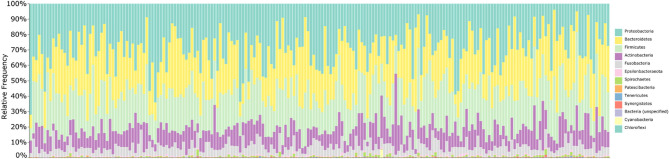
Taxonomic summary of saliva samples. Bar chart summarizing the taxonomic composition of each saliva sample at the phylum level. Bars are ordered by participant age; taxonomy was assigned to 16S V4 amplicon sequences using the SILVA 132 database. Overall, samples are dominated by the phyla *Proteobacteria, Bacteroidetes, Firmicutes, Actinobacteria*, and *Fusobacteria*.

Following removal of putative contaminant features, we summarized the taxonomic composition of the 205 saliva samples that formed the core of our microbiota analysis (Figure 1). The samples were dominated by the phyla *Proteobacteria* (30%), *Bacteroidetes* (26%), *Firmicutes* (25%), *Actinobacteria* (12%), and *Fusobacteria* (6%), with sequences assigned to *Epsilonbacteraeoata, Spirochaetes, Patescibacteria, Tenericutes, Synergistetes, Cyanobacteria, Chloroflexi*, and unassigned *Bacteria* making up the remaining 1%. At the genus level, the salivary microbiota was dominated by *Prevotella* (18%), *Neisseria* (14%), *Haemophilus* (12%), *Streptococcus* (9%), *Rothia* (8%), *Veillonella* (6%), *Fusobacterium* (4%), *Alloprevotella* (3%), *Porphyromonas* (3%), *Gemella* (2%), *Granulicatella* (2%), *Leptotrichia* (2%), *Actinomyces* (2%), and *Aggregatibacter* (2%), with various genera accounting for 1% or less of total sequences making up the remaining 13%. Taxonomic assignment of sequences using HOMD in place of SILVA yielded nearly identical classifications (Supplementary Table 4).

### Age Significantly Impacts Salivary Microbiota in NPA Children

**Figure 2:**
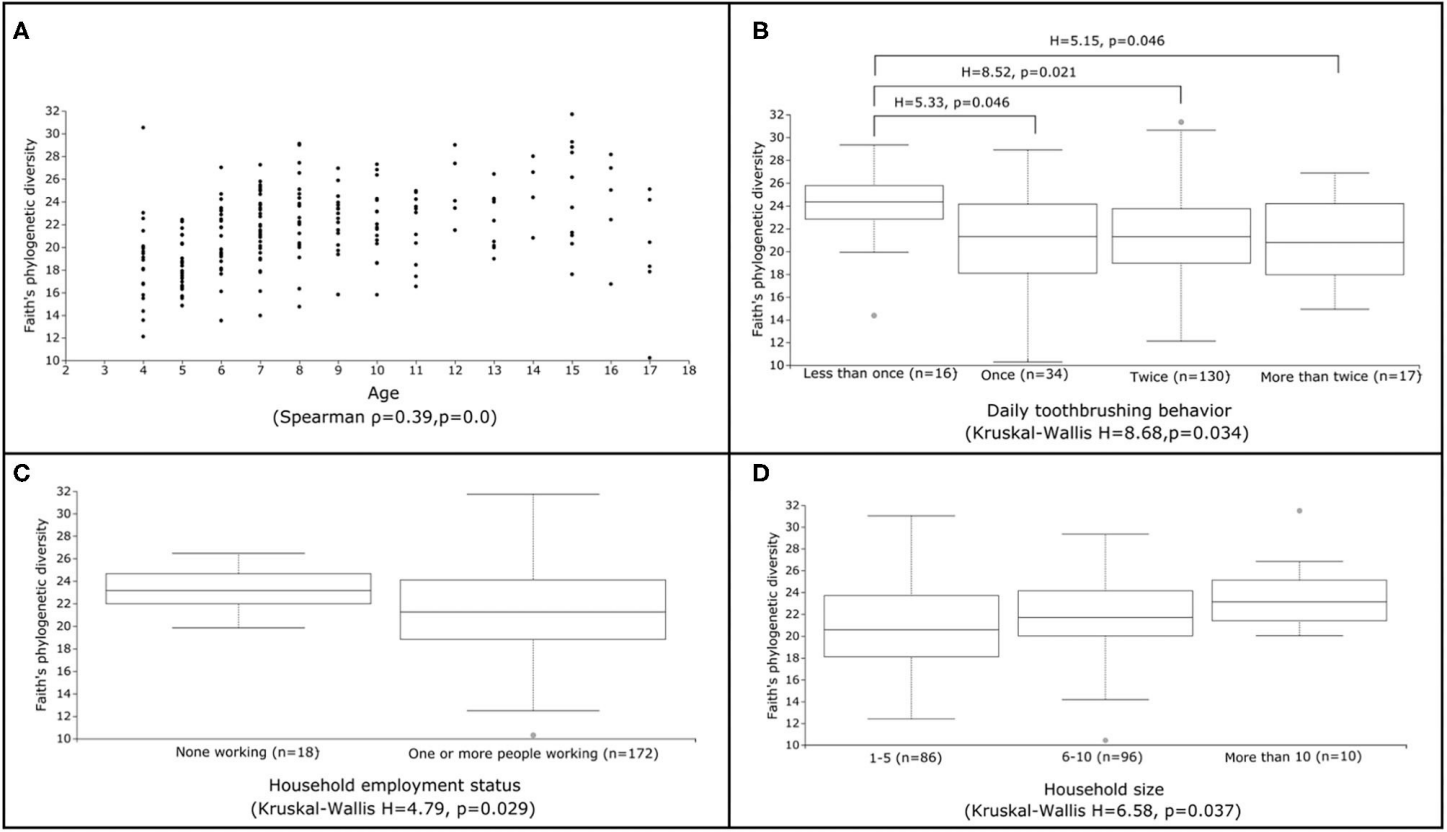
Factors linked to significant differences in alpha diversity. Scatter **(A)** and box **(B-D)** plots illustrating the range of Faith's phylogenetic diversity values for selected metadata categories identified as significantly linked to alpha diversity (**A**: age, **B**: daily toothbrushing behavior, **C**: household employment status, **D**: household size). Results of overall Spearman correlation (continuous variables) and Kruskal-Wallis (categorical variables) tests are also displayed, along with any statistically significant (FDR *p* <0.05) pairwise Kruskal-Wallis test comparisons between groups. All other pairwise Kruskal-Wallis test comparisons were not statistically significant.

We tested all available metadata factors (Supplementary Table 1) for associations with changes in microbial diversity (alpha diversity) (Table 2; Figure 2), composition (beta diversity) (Table 2) and abundance of microbial features (Table 3). Participant age (Spearman ρ = 0.4, *p* = 0.0; *R*^2^ = 0.048, *p* = 0.001) and examination date (H = 26.7, *p* = 8 ×10^−4^; *R*^2^ = 0.101, *p* = 0.001) were significantly related to both diversity and composition (Table 2). A feature classified as uncultured *Actinomyces* [identified as *Actinomyces* using HOMD (Supplementary Table 6)] was significantly associated with examination date (Table 3); this feature was observed on 16 of the 26 different examination dates and varied in abundance. Additionally, salivary pH and flow rate were significantly associated with microbiota diversity (Spearman ρ = 0.18, *p* = 0.011 and ρ = 0.28, *p* = 1 ×10^−4^, respectively) and composition (*R*^2^ = 0.013, *p* = 0.007 and *R*^2^ = 0.028, *p* = 0.001) (Table 2). However, multi-factor adonis tests indicated that both examination date (*R*^2^ = 0.063, *p* = 0.025) and salivary flow rate (*R*^2^ = 0.013, *p* = 0.005) were confounded with age although examination date retained some independent explanatory power (R^2^ = 0.075, *p* = 0.001). Salivary pH remained independently significant in the multi-factor test (*R*^2^ = 0.011, *p* = 0.014) (Table 2). We accounted for age in subsequent beta diversity (i.e., composition) analyses using multi-factor adonis tests (Table 2), and report only age-adjusted results (where applicable) in the remainder of the text.

**Table 2:** Metadata factors linked to significant differences in alpha and beta diversity across sample groups.

**Alpha diversity (Faith's phylogenetic diversity)**						
**Category**		**Spearman correlation test results**				
Age		0.39	0.0			
Saliva pH		0.18	0.011			
Saliva flow rate		0.28	1 × 10^−4^			
Total carious surfaces		0.2	0.005			
		**Kruskal-Wallis test results**				
		**H**	***P***			
Daily toothbrushing		8.68	0.034			
Examination date		26.7	8 × 10^−4^			
Household employment status		4.79	0.029			
Household size		6.58	0.037			
**Household employment status**	**Household employment status**		**Household employment status**		**Household employment status**	
Age	0.046	0.001	NA	NA	0.043	0.001
Examination date	0.101	0.001	0.075	0.001	0.093	0.001
Saliva pH	0.013	0.007	0.011	0.014	0.015	0.004
Saliva flow rate	0.028	0.001	0.006	0.23	0.025	0.001
Soft drink consumption	0.008	0.05	0.007	0.067	0.008	0.074
Household size	0.019	0.011	0.015	0.054	0.016	0.043
Household employment status	0.012	0.013	0.011	0.009	0.012	0.004
Caries status	0.009	0.039	0.008	0.052	NA	NA
Caries severity	0.025	0.007	0.02	0.034	NA	NA
Total carious surfaces	0.025	0.001	0.014	0.002	0.023	0.001

*To test for significant differences in alpha diversity, a Spearman correlation test was used for numerical metadata categories; Kruskal-Wallis tests were used for categorical metadata categories. To test for significant differences in beta diversity, adonis tests were used for both numerical and categorical metadata categories. The results of each significant test for both alpha and beta diversity are displayed; categories that did not return a significant result for a given test are excluded fromthe relevant section of the table*.

**Table 3:** Microbial features differing significantly in abundance across groups identified by ANCOM.

**Category**	**Feature Taxonomy**	**ANCOM W-value**	**Prevalence (no. of samples detected in)**	**Abundance (no. of sequences across all samples)**	**Group association**
Daily toothbrushing	*Streptococcus sobrinus*	279	51	433	Brush teeth less than once per day
Caries status	*Actinobacillus porcinus*	81	5	8,649	Caries-free
	*Leptotrichia*	26	11	121	Caries-free
	*Veillonella* uncultured organism	19	195	24,662	Caries-active
Caries severity	*Lactobacillus gasseri*	208	44	1,660	Severe caries
Examination date	*Actinomyces* uncultured bacterium	622	59	3,978	Unclear

*Specific microbial features significantly associated with daily toothbrushing behavior, caries status, caries severity, and examination date are listed below. The metadata category, brief feature taxonomy as assigned using the SILVA 132 database, the reported W-value from ANCOM testing, overall prevalence (i.e., number of samples the feature was detected in), overall abundance (i.e., total number of sequences associated with the feature), and group association (the sample group in which the feature was most abundant) are displayed for each significant feature. Samples with unknown or unrecorded values for a given category were removed prior to ANCOM testing. Individual IDs and full taxonomy strings for significant features are given in Supplementary Table 5; comparison of taxonomic classification of the significant features using the SILVA and HOMD databases is given in Supplementary Table 6*.

### Behavioral Factors Impact Salivary Microbiota Diversity and Composition

We investigated associations between the salivary microbiota and behavioral factors known to impact caries risk. The number of times per day a child reported brushing their teeth was significantly associated with alpha diversity (H = 8.68, *p* = 0.034) (Table 2). Children who reported brushing their teeth less than once per day harbored higher diversity and exhibited less inter-individual variation in alpha diversity than children who reported more frequent tooth brushing (Figure 2). A feature classified as *Streptococcus sobrinus* was significantly more abundant in the saliva of children who reported brushing their teeth less than once per day (Table 3). However, tooth brushing was not associated with significant change in overall composition. Of dietary variables collected, only self-reported soft (i.e., carbonated) drink consumption approached a significant association with microbiota composition (*R*^2^ = 0.007, *p* = 0.067), an effect that may have been confounded by other factors such as age and caries severity (Table 2).

### Socioeconomic Factors Are Linked to Salivary Microbiota

We used self-reported questionnaire data on household size and employment status in the child's household as proxies for socioeconomic status (SES). Employment status was significantly related to alpha diversity (H = 4.79, *p* = 0.029), with children who reported no people in their household working having higher average diversity and lower inter-individual variability than those who reported at least one person working (Table 2; Figure 2). Household size was also generally associated with higher average diversity (H = 6.58, *p* = 0.037) (Table 2; Figure 2); however, no pairwise significant differences in diversity were observed between groups. Both employment status (*R*^2^ = 0.011, *p* = 0.009; permdisp F = 8.68, *p* = 0.008) and household size (*R*^2^ = 0.015, *p* = 0.054) were significantly related to microbiota composition (Table 2). Specifically, significant differences in composition were found between children who reported living with 1–5 people and those who reported living with 6–10 (pairwise PERMANOVA FDR-corrected *p* = 0.039) or more than 10 people (pairwise PERMANOVA FDR-corrected *p* = 0.041). However, ANCOM testing did not identify any microbial features that changed significantly in abundance across these groups.

### Dental Caries Is Associated With Salivary Microbiota Composition

Finally, we explored associations between caries and the salivary microbiota. Total number of carious surfaces was significantly associated with alpha diversity (Spearman ρ = 0.2, *p* = 0.005) (Table 2). Variations in composition were associated with caries status (i.e., caries-free or caries-active) (*R*^2^ = 0.008, *p* = 0.052), caries severity (*R*^2^ = 0.02, *p* = 0.034), and number of carious surfaces (*R*^2^ = 0.014, *p* = 0.002) (Table 2). Pairwise tests comparing the caries-free or incipient caries groups with the moderate or severe caries groups approached significance (pairwise PERMANOVA FDR-corrected *p* = 0.072). We used ANCOM differential abundance testing to identify microbial features driving these compositional changes (Table 3). The *Actinobacillus porcinus* feature previously associated with examination date was significantly associated with caries status and was more abundant in the caries-free group (W = 81) (Table 3). Members of the genera *Leptotrichia* (W = 26) and *Veillonella* (W = 19) were significantly more abundant in the caries-free and caries-active groups, respectively (Table 3). A microbial feature identified as *Lactobacillus gasseri* was significantly associated with caries severity (W = 208), specifically with the severe caries group (Table 3).

We further sought to understand whether caries severity interacted with other factors significantly or near-significantly related to salivary microbiota composition using multi-factor adonis tests (Table 2). Caries severity consistently explained 2.1–3.2% of variation in the dataset and was significantly associated with composition (*p* <0.05). Self-reported soft drink consumption co-varied with caries severity (*R*^2^ = 0.008, *p* = 0.074) (Table 2). However, saliva pH (*R*^2^ = 0.015, *p* = 0.004), saliva flow rate (*R*^2^ = 0.025, *p* = 0.001), household size (*R*^2^ = 0.016, *p* = 0.043) household employment status (*R*^2^ = 0.012, *p* = 0.004), and total carious surfaces (*R*^2^ = 0.023, *p* = 0.001) were significantly related to composition, independent of caries severity (Table 2).

## Discussion

This is the first study to examine the biological, behavioral, and socioeconomic factors driving overall salivary microbiota diversity and composition in Aboriginal and Torres Strait Islander children and adolescents. Although Aboriginal and Torres Strait Islander children, especially those living in remote communities, are at high risk of caries [3–5, 10], little is known about the role of the oral microbiota and how it may mediate disease in this group [50]. While several recent studies have investigated oral microbiota in Indigenous individuals around the world, many have focused primarily on characterizing differences between human groups living different lifestyles [26–28]. Only a handful of studies have examined links between oral microbiota and oral health in Indigenous populations [13, 50]. In our study, we found that salivary microbiota diversity in Aboriginal and Torres Strait Islander children and adolescents from the NPA is linked to age, salivary characteristics, number of carious surfaces, toothbrushing behaviors, and SES (Table 2; Figure 2). The composition of salivary microbiota is related to age, salivary characteristics, self-reported soft drink consumption, SES, and dental caries (Table 2). We identified microbial features that significantly varied in abundance according to examination date, toothbrushing behavior, caries status, and caries severity (Table 3). We acknowledge that the number of caries-active individuals in this study clearly outweighs the caries-free group; however, this was a population-based study, not focused on the recruitment of caries-active children. Accordingly, our results should be interpreted to identify these factors within a population where caries is highly prevalent. While saliva does not represent a singular, structured oral microbial community, it is easy and non-invasive to collect and provides a broad overview of the microbes present in the oral cavity [51]. Our findings contribute to a novel understanding of the mechanistic associations between biological, behavioral and socioeconomic factors, the oral microbiota, and dental caries in this population of children, with implications for the microbiota-aware treatment and prevention of oral diseases.

Behavioral factors, such as toothbrushing frequency and soft drink consumption, were linked to changes in microbiota diversity and composition in our dataset. The ability of microbes to grow at a given site depends on environmental factors, such as pH and oxygen or nutrient availability, which may be altered by host behavior. Less frequent brushing was linked to higher microbial alpha diversity in our study (Figure 2). Regular tooth brushing interrupts the accumulation of microbial species on the surfaces of the oral cavity and thereby lowers the overall diversity of microbes found in saliva. Further, a feature classified as *Streptococcus sobrinus* was significantly more abundant in the salivary microbiota of children who reported brushing their teeth less than once per day (Table 3). *S. sobrinus* has long been associated with dental caries in the literature and is thought to aggravate caries when found in association with other cariogenic species such as *Streptococcus mutans* [52]. Of interest, a recent study of supragingival plaque microbiota in non-Indigenous Australian children also reported that *Streptococcus* abundance decreased according to tooth brushing frequency [53]. However, *S. mutans* itself was not significantly associated with dental caries or other metadata factors in our microbiota analysis. Soft drink consumption, which was widespread within our study population (Table 1), decreases environmental pH in the mouth through the introduction of free sugars that microbes ferment to acid [11]. The established impact of environmental pH on oral microbial communities is further supported by our result that salivary pH was significantly associated with salivary microbiota composition and diversity (Table 2). Overall, our findings suggest that daily behavioral activities linked to caries development can impact child salivary microbiota in this population. Understanding which microbes respond to specific factors such as tooth brushing could be translated into personalized medical approaches in the future, providing tailored recommendations relevant to the individual's microbiota composition.

We also found that socioeconomic factors, such as household size and employment status of household members, were associated with salivary microbiota diversity and composition in NPA children (Table 2). It is well-established that low SES increases caries risk in children [3, 8, 10]. However, whether microbial mechanisms mediate this risk is less well-understood. Johansson et al. demonstrated differences in dental plaque microbiota richness and composition between a low-SES, high-caries population in Romania and a high-SES, low-caries population in Sweden [8]. This comparison is confounded by large cultural, geographic, and historical differences, so it is of interest that we, for the first time, demonstrate that a similar pattern in salivary microbiota in a comparatively homogeneous population. Here, children who reported no one in their household working had less variability in microbial composition compared to the group who reported at least one person working. However, given that household size co-varied with caries severity in our dataset and that significant differences in dispersion linked to household employment status were identified by permdisp testing, these findings require further investigation to determine whether these factors are mechanistically linked to caries.

In our study population, multiple measures of caries were significantly associated with salivary microbiota composition (Table 2), supporting previous findings that salivary microbiota correlates with caries status [14]. In particular, pairwise comparisons of the caries-free or incipient caries groups to the moderate or severe caries groups approached significance, suggesting that progression of caries to more advanced stages may involve a distinct shift in microbiota composition. Studies of dental plaque have identified shifts in microbial community composition related to caries progression [13, 15, 54]; such patterns may be less obvious, but still present, when sampling saliva rather than tooth surfaces [55, 56]. A feature classified as *Lactobacillus gasseri* was significantly associated with caries severity in our dataset and was most abundant in the severe caries group (Table 3). *L. gasseri* has previously been identified in the human mouth [57]; as a lactic acid bacterium, it likely participates in sugar fermentation and hence, caries promotion. A *Veillonella* feature was significantly more abundant in caries-active samples, while features classified as *Leptotrichia* and *Actinobacillus porcinus* were more abundant in the caries-free group (Table 3). Numerous studies have reported that *Veillonella* or *Veillonellaceae* species are associated with dental caries [12–14, 16, 54]. *Veillonella* species use lactic acid as their primary energy source and therefore are closely associated with caries-promoting species that produce lactate [12, 58]. Because of this association, *Veillonella* levels in plaque and saliva have been suggested as a biomarker for future caries risk even at apparently healthy sites [16, 54, 58]. Of interest, a *Veillonella* species was previously identified as significantly more abundant in the dental plaque microbiota of Canadian First Nations children with early childhood caries compared to caries-free counterparts [13]. The association of *Leptotrichia* species with oral health and disease is less clear; some species in this genus may be disease-associated and others health-associated [8, 54]. The importance of the *Actinobacillus porcinus* feature in oral health is also difficult to interpret, as this species is not typically found in the human oral microbiota. However, the 16S amplicon sequence associated with this feature was classified as *Haemophilus* using the Human Oral Microbiome Database (Supplementary Table 6). Other recent publications have reported that *Haemophilus* is found in higher relative abundance in the saliva of caries-free children and adults compared to those with caries [56, 59], although this association is not universal [13]. Better characterization of the oral health relevance of *Leptotrichia* and *Haemophilus* strains present in the NPA child population could be useful in understanding microbial oral health and informing new therapeutic strategies.

While this study is the first of its kind, there are several limitations. While our study used saliva samples to profile the oral microbiota, samples of plaque biofilm from specific tooth sites might reveal closer associations between the microbiota and disease [51, 60]. However, plaque collection was not practicable under the field conditions of our study. Saliva samples, especially after a period of chewing wax, which would dislodge much adherent biofilm [61], give an overview of oral microbiota [51, 55, 60, 62] and can be related to overall caries experience and activity of the individual child. In addition, some metadata information was collected using self-administered questionnaires, data on last meal before sample collection was not collected, and our dataset lacked sampling controls to detect contamination at the sampling site. In relation to this last point, investigation of sampling controls by our group in later years of the clinical trial demonstrated minimal overlap between saliva and sampling controls, indicating negligible contamination of saliva during sampling [50]. We also saw that some significant factors co-varied, making it difficult to distinguish the specific mechanisms underpinning each factor, i.e., whether these factors work together or independently to influence microbiota composition, or are both correlated with some other, unmeasured factor. For example, examination date was significant in explaining variation in both alpha and beta diversity (Table 2) and was associated with a significant change in abundance of a specific *Actinomyces* feature (Table 3). However, further investigation suggested that examination date was partially confounded with age, meaning that these associations may not be directly linked to examination date but an artifact of collecting samples from children of different ages at different schools on different days. In another example, we found that soft drink consumption co-varied with caries severity (Table 2), making it difficult to discern whether either of these factors acts independently. Further research into each of these factors is needed to better understand their contributions to oral microbiota diversity and composition.

For the first time, we describe the salivary microbiota of Aboriginal and Torres Strait Islander children living in a remote location with limited access to dental care. We identify relationships between the salivary microbiota, dental caries, and known caries risk factors such as behavioral activities and SES. Given the importance of the oral microbiota for oral health, refining our understanding of oral microbial communities and how they mediate oral health and disease could be key to informing better treatment and prevention strategies, particularly in populations at high risk of oral disease. This understanding may be especially important for the oral health of Australian Aboriginal and Torres Strait Islander peoples, as early evidence suggests a distinctive relationship between these peoples and their associated oral microbes [29]. Datasets such as ours form a baseline for longitudinal studies of caries prevention and will be key in ensuring that new microbiome-based or microbiome-aware therapies are also applicable to Indigenous communities and do not damage or disrupt Indigenous microbiota. Future research toward this goal could include the investigation of different sample types, such as dental plaque, that allow for a more structured view of oral microbial communities; inclusion of more Aboriginal and Torres Strait Islander communities across Australia; employment of more precise sequencing techniques, such as shotgun metagenomics, to obtain species-level identification of oral microbes; and collection of more detailed metadata to support a finer-scale understanding of the relationship between oral microbiota and, for example, diet.

## Data Availability Statement

The datasets presented in this article are not readily available because of ethical and privacy constraints and respect for principles of Indigenous data sovereignty. Data may be made available for further analyses on a case-by-case basis subject to HREC review and community approval. Requests to access the datasets should be directed to Newell W. Johnson, n.johnson@griffith.edu.au.

## Ethics Statement

The studies involving human participants were reviewed and approved by Griffith University Human Research Ethics Committee, Far North Queensland (FNQ) Human Research Ethics Committee, Department of Education and Training (Queensland Government), and Torres and Cape Hospital and Health Service. Written informed consent to participate in this study was provided by the participants' legal guardian/next of kin.

## Author Contributions

MH-D performed data analysis and interpretation and wrote the manuscript. ES performed lab work and data processing and critically revised the manuscript. NJ contributed to study conception and design, data acquisition and interpretation, and critically revised the manuscript. KK and RL contributed to data acquisition and critically revised the manuscript. JK contributed to study conception and design, ethical approvals, data acquisition, and critically revised the manuscript. LW contributed to study conception and design, contributed to data interpretation, and critically revised the manuscript. All authors gave their final approval and agree to be accountable for all aspects of the work.

## Conflict of Interest

The authors declare that the research was conducted in the absence of any commercial or financial relationships that could be construed as a potential conflict of interest.

## References

[1] Gilchrist F Marshman Z Deery C Rodd HD. The impact of dental caries on children and young people: what they have to say? Int J Paediatr Dent. (2015) 25:327–38. 10.1111/ipd.12186 26153526

[2] Butten K Johnson NW Hall KK Toombs M King N O'Grady K-AF. Impact of oral health on Australian urban Aboriginal and Torres Strait Islander families: a qualitative study. Int J Equity Health. (2019) 18:34. 10.1186/s12939-019-0937-y 30777079 PMC6378750

[3] Ha DH Roberts-Thomson KF Arrow P Peres KG Do LG. Children's oral health status in Australia, 2012-2014. In: Do LG, Spencer AJ, editors. National Survey of Child Oral Health 2012-2014. Adelaide: University of Adelaide Press (2016). p. 86–152.

[4] Lalloo R Kroon J Tut O Kularatna S Jamieson LM Wallace V. Effectiveness, cost-effectiveness and cost-benefit of a single annual professional intervention for the prevention of childhood dental caries in a remote rural Indigenous community. BMC Oral Health. (2015) 15:99. 10.1186/s12903-015-0076-9 26318162 PMC4553010

[5] Roberts-Thomson KF Kapellas K Ha D Jamieson LM Arrow P Do LG. Oral health status and behaviours of Indigenous Australian children. In: Do LG, Spencer AJ, editors. Oral health of Australian children: The National Child Oral Health Study 2012–14. Adelaide: University of Adelaide Press (2016). p. 264–87.

[6] Hopcraft M Chow W. Dental caries experience in aboriginal and torres strait islanders in the Northern Peninsula Area, Queensland. Aust Dent J. (2007) 52:300–4. 10.1111/j.1834-7819.2007.tb00506.x 18265686

[7] Kroon J Lalloo R Tadakamadla SK Johnson NW. Dental caries experience in children of a remote Australian Indigenous community following passive and active preventive interventions. Community Dent Oral Epidemiol. (2019) 47: 1−7. 10.1111/cdoe.12486 PMC6899803 31328295

[8] Johansson I Witkowska E Kaveh B Lif Holgerson P Tanner ACR. The microbiome in populations with a low and high prevalence of caries. J Dent Res. (2016) 95:80–6. 10.1177/0022034515609554 26442950 PMC4700664

[9] Fernando S Kumar S Bakr M Speicher D Lea R Scuffham PA. Children's untreated decay is positively associated with past caries experience and with current salivary loads of mutans Streptococci; negatively with self-reported maternal iron supplements during pregnancy: a multifactorial analysis. J Public Health Dent. (2019) 79:109–15. 10.1111/jphd.12301 30551255

[10] Butten K Johnson N Anderson J Toombs M King N O'Grady K. Risk factors for oral health in young, urban, Aboriginal and Torres Strait Islander children. Aust Dent J. (2019) 64:72–81. 10.1111/adj.12662 30375649 PMC6392135

[11] Tanner ACR Kressirer CA Rothmiller S Johansson I Chalmers NI. The caries microbiome: implications for reversing dysbiosis. Adv Dent Res. (2018) 29:78–85. 10.1177/0022034517736496 29355414

[12] Gross EL Beall CJ Kutsch SR Firestone ND Leys EJ Griffen AL. Beyond Streptococcus mutans: dental caries onset linked to multiple species by 16S rRNA community analysis. PLoS ONE. (2012) 7:e47722. 10.1371/journal.pone.0047722 23091642 PMC3472979

[13] Agnello M Marques J Cen L Mittermuller B Huang A Chaichanasakul Tran N. Microbiome associated with severe caries in Canadian first nations children. J Dent Res. (2017) 96:1378–85. 10.1177/0022034517718819 28709393 PMC5652857

[14] Eriksson L Holgerson PL Johansson I. Saliva and tooth biofilm bacterial microbiota in adolescents in a low caries community. Sci Rep. (2017) 7:5861. 10.1038/s41598-017-06221-z 28724921 PMC5517611

[15] Espinoza JL Harkins DM Torralba M Gomez A Highlander SK Jones MB. Supragingival plaque microbiome ecology and functional potential in the context of health and disease. mBio. (2018) 9:e01631–18. 10.1128/mBio.01631-18 30482830 PMC6282201

[16] Xu L Chen X Wang Y Jiang W Wang S Ling Z. Dynamic alterations in salivary microbiota related to dental caries and age in preschool children with deciduous dentition: a 2-year follow-up study. Front Physiol. (2018) 9:342. 10.3389/fphys.2018.00342 29670544 PMC5893825

[17] Blekhman R Goodrich JK Huang K Sun Q Bukowski R Bell JT. Host genetic variation impacts microbiome composition across human body sites. Genome Biol. (2015) 16:191. 10.1186/s13059-015-0759-1 26374288 PMC4570153

[18] Corby PM Bretz WA Hart TC Schork NJ Wessel J Lyons-Weiler J. Heritability of oral microbial species in caries-active and caries-free twins. Twin Res Hum Genet. (2007) 10:821–8. 10.1375/twin.10.6.821 18179393 PMC3148892

[19] Demmitt BA Corley RP Huibregtse BM Keller MC Hewitt JK McQueen MB. Genetic influences on the human oral microbiome. BMC Genomics. (2017) 18:659. 10.1186/s12864-017-4008-8 28836939 PMC5571580

[20] Gomez A Espinoza JL Harkins DM Leong P Saffery R Bockmann M. Host genetic control of the oral microbiome in health and disease. Cell Host Microbe. (2017) 22:269–78.e3. 10.1016/j.chom.2017.08.013 28910633 PMC5733791

[21] Hansen TH Kern T Bak EG Kashani A Allin KH Nielsen T. Impact of a vegan diet on the human salivary microbiota. Sci Rep. (2018) 8:5847. 10.1038/s41598-018-24207-3 29643500 PMC5895596

[22] Kato I Vasquez A Moyerbrailean G Land S Djuric Z Sun J. Nutritional correlates of human oral microbiome. J Am Coll Nutr. (2017) 36:88–98. 10.1080/07315724.2016.1185386 27797671 PMC5477991

[23] Marsh PD. Microbiology of dental plaque biofilms and their role in oral health and caries. Dent Clin North Am. (2010) 54:441–54. 10.1016/j.cden.2010.03.002 20630188

[24] Weyrich LS Duchene S Soubrier J Arriola L Llamas B Breen J. Neanderthal behaviour, diet, and disease inferred from ancient DNA in dental calculus. Nature. (2017) 544:357–61. 10.1038/nature21674 28273061

[25] Handsley-Davis M Jamieson L Kapellas K Hedges J Weyrich LS. The role of the oral microbiota in chronic non-communicable disease and its relevance to the Indigenous health gap in Australia. BMC Oral Health. (2020) 20:327. 10.1186/s12903-020-01308-y 33198712 PMC7670664

[26] Clemente JC Pehrsson EC Blaser MJ Sandhu K Gao Z Wang B. The microbiome of uncontacted Amerindians. Sci Adv. (2015) 1:e1500183. 10.1126/sciadv.1500183 26229982 PMC4517851

[27] Lassalle F Spagnoletti M Fumagalli M Shaw L Dyble M Walker C. Oral microbiomes from hunter-gatherers and traditional farmers reveal shifts in commensal balance and pathogen load linked to diet. Mol Ecol. (2018) 27:182–95. 10.1111/mec.14435 29165844

[28] Nasidze I Li J Schroeder R Creasey JL Li M Stoneking M. High diversity of the saliva microbiome in batwa pygmies. PLoS ONE. (2011) 6:e23352. 10.1371/journal.pone.0023352 21858083 PMC3156759

[29] Handsley-Davis M. *Investigating Oral Microbial Communities in Aboriginal Australians* (BSc. Honours thesis). Australia: University of Adelaide (2016).

[30] Lalloo R Tadakamadla SK Kroon J Jamieson LM Ware RS Johnson NW. Carious lesions in permanent dentitions are reduced in remote Indigenous Australian children taking part in a non-randomised preventive trial. PLoS ONE. (2021) 16:e0244927. 10.1371/journal.pone.0244927 33507984 PMC7842954

[31] Gugnani N Pandit I Srivastava N Gupta M Sharma M. International Caries Detection and Assessment System (ICDAS): a new concept. Int J Clin Pediatr Dent. (2011) 4:93–100. 10.5005/jp-journals-10005-1089 27672245 PMC5030492

[32] Stevens K. Developing a descriptive system for a new preference-based measure of health-related quality of life for children. Qual Life Res. (2009) 18:1105–13. 10.1007/s11136-009-9524-9 19693703

[33] Slade GD Spencer AJ. Development and evaluation of the oral health impact profile. Community Dent Health. (1994) 11:3–11. 8193981

[34] Caporaso JG Lauber CL Walters WA Berg-Lyons D Huntley J Fierer N. Ultra-high-throughput microbial community analysis on the Illumina HiSeq and MiSeq platforms. ISME J. (2012) 6:1621–4. 10.1038/ismej.2012.8 22402401 PMC3400413

[35] Bolyen E Rideout JR Dillon MR Bokulich NA Abnet CC Al-Ghalith GA. Reproducible, interactive, scalable and extensible microbiome data science using QIIME 2. Nat Biotechnol. (2019) 37:852–7. 10.1038/s41587-019-0209-9 31341288 PMC7015180

[36] Amir A McDonald D Navas-Molina JA Kopylova E Morton JT Xu ZZ. Deblur rapidly resolves single-nucleotide community sequence patterns. mSystems. (2017) 2:e00191–16. 10.1128/mSystems.00191-16 28289731 PMC5340863

[37] Bisanz JE.qiime2R: Importing QIIME2 Artifacts and Associated Data into R Sessions R GitHub (2018).

[38] Davis NM Proctor DM Holmes SP Relman DA Callahan BJ. Simple statistical identification and removal of contaminant sequences in marker-gene and metagenomics data. Microbiome. (2018) 6:226. 10.1186/s40168-018-0605-2 30558668 PMC6298009

[39] Faith DP Baker AM. Phylogenetic diversity (PD) and biodiversity conservation: some bioinformatics challenges. Evol Bioinforma Online. (2007) 2:121–8. 10.1177/117693430600200007 19455206 PMC2674678

[40] Lozupone C Knight R. UniFrac: a new phylogenetic method for comparing microbial communities. Appl Environ Microbiol. (2005) 71:8228–35. 10.1128/AEM.71.12.8228-8235.2005 16332807 PMC1317376

[41] Kruskal WH Wallis WA. Use of ranks in one-criterion variance analysis. J Am Stat Assoc. (1952) 47:583–621. 10.1080/01621459.1952.10483441

[42] Anderson MJ. A new method for non-parametric multivariate analysis of variance. Austral Ecol. (2001) 26:32–46. 10.1046/j.1442-9993.2001.01070.x

[43] Oksanen J Blanchet FG Friendly M Kindt R Legendre P McGlinn D. vegan: Community Ecology Package CRAN (2018).

[44] Anderson MJ. Distance-based tests for homogeneity of multivariate dispersions. Biometrics. (2006) 62:245–53. 10.1111/j.1541-0420.2005.00440.x 16542252

[45] Mandal S Van Treuren W White RA Eggesbø M Knight R Peddada SD. Analysis of composition of microbiomes: a novel method for studying microbial composition. Microb Ecol Health Dis. (2015) 26:27663. 10.3402/mehd.v26.27663 26028277 PMC4450248

[46] RStudio Team. RStudio: Integrated development for R. Boston, MA: RStudio, Inc. (2015).

[47] Wickham H. ggplot2: Elegant Graphics for Data Analysis. New York, NY: Springer-Verlag (2009).

[48] Eisenhofer R Minich JJ Marotz C Cooper A Knight R Weyrich LS. Contamination in low microbial biomass microbiome studies: issues and recommendations. Trends Microbiol. (2019) 27:105–17. 10.1016/j.tim.2018.11.003 30497919

[49] Weyrich LS Farrer AG Eisenhofer R Arriola LA Young J Selway CA. Laboratory contamination over time during low-biomass sample analysis. Mol Ecol Resour. (2019) 19:982–96. 10.1111/1755-0998.13011 30887686 PMC6850301

[50] Skelly E Johnson NW Kapellas K Kroon J Lalloo R Weyrich L. Response of salivary microbiota to caries preventive treatment in aboriginal and torres strait islander children. J Oral Microbiol. (2020) 12:1830623. 10.1080/20002297.2020.1830623 33149844 PMC7586720

[51] Eren AM Borisy GG Huse SM Welch JLM. Oligotyping analysis of the human oral microbiome. Proc Natl Acad Sci USA. (2014) 111:E2875–84. 10.1073/pnas.1409644111 24965363 PMC4104879

[52] Okada M Kawamura M Oda Y Yasuda R Kojima T Kurihara H. Caries prevalence associated with *Streptococcus mutans* and *Streptococcus sobrinus* in Japanese schoolchildren. Int J Paediatr Dent. (2012) 22:342–8. 10.1111/j.1365-263X.2011.01203.x 22225789

[53] Freire M Moustafa A Harkins DM Torralba MG Zhang Y Leong P. Longitudinal study of oral microbiome variation in twins. 1. Sci Rep. (2020) 10:7954. 10.1038/s41598-020-64747-1 32409670 PMC7224172

[54] Richards VP Alvarez AJ Luce AR Bedenbaugh M Mitchell ML Burne RA. Microbiomes of site-specific dental plaques from children with different caries status. Infect Immun. (2017) 85:e00106–17. 10.1128/IAI.00106-17 28507066 PMC5520424

[55] Teng F Yang F Huang S Bo C Xu ZZ Amir A. Prediction of early childhood caries via spatial-temporal variations of oral microbiota. Cell Host Microbe. (2015) 18:296–306. 10.1016/j.chom.2015.08.005 26355216

[56] Belstrøm D Holmstrup P Fiehn N-E Kirkby N Kokaras A Paster BJ. Salivary microbiota in individuals with different levels of caries experience. J Oral Microbiol. (2017) 9:1270614. 10.1080/20002297.2016.1270614 28326153 PMC5328370

[57] Human Oral Microbiome Database (HOMD). Lactobacillus gasseri Human Oral Microbiome Taxon Description.

[58] Mashima I Nakazawa F. Interaction between Streptococcus spp. and *Veillonella tobetsuensis* in the early stages of oral biofilm formation. J Bacteriol. (2015) 197:2104–11. 10.1128/JB.02512-14 25917902 PMC4455269

[59] Chen W Jiang Q Yan G Yang D. The oral microbiome and salivary proteins influence caries in children aged 6 to 8 years. BMC Oral Health. (2020) 20:295. 10.1186/s12903-020-01262-9 33115458 PMC7592381

[60] Segata N Haake SK Mannon P Lemon KP Waldron L Gevers D. Composition of the adult digestive tract bacterial microbiome based on seven mouth surfaces, tonsils, throat and stool samples. Genome Biol. (2012) 13:R42. 10.1186/gb-2012-13-6-r42 22698087 PMC3446314

[61] Dawes C Tsang RWL Suelzle T. The effects of gum chewing, four oral hygiene procedures, and two saliva collection techniques, on the output of bacteria into human whole saliva. Arch Oral Biol. (2001) 46:625–32. 10.1016/S0003-9969(01)00017-6 11369317

[62] Baca P Castillo AM Baca AP Liébana MJ Junco P Liébana J. Genotypes of *Streptococcus mutans* in saliva versus dental plaque. Arch Oral Biol. (2008) 53:751–4. 10.1016/j.archoralbio.2008.02.011 18374899

