# Supplementary material for: Biocultural Drivers of Salivary Microbiota in Australian Aboriginal and Torres Strait Islander Children
Source: Front Oral Health. 2021 Mar 18;2:641328. doi: 10.3389/froh.2021.641328 (PMC8757737; doi:10.3389/froh.2021.641328)
Supplement: Supplementary file 1 [file Data_Sheet_1.docx]

Supplementary Material

**Supplementary Figure 1. Tracking contamination in saliva samples.** Principal coordinates analysis (PCoA) of unweighted UniFrac distances between saliva samples and negative controls (EBCs and NTCs), rarefied to 500 sequences per sample (A). A scatter plot showing prevalence of each microbial feature in saliva samples and negative controls is displayed in (B). Features more prevalent in negative controls than in samples are considered contaminants.

**
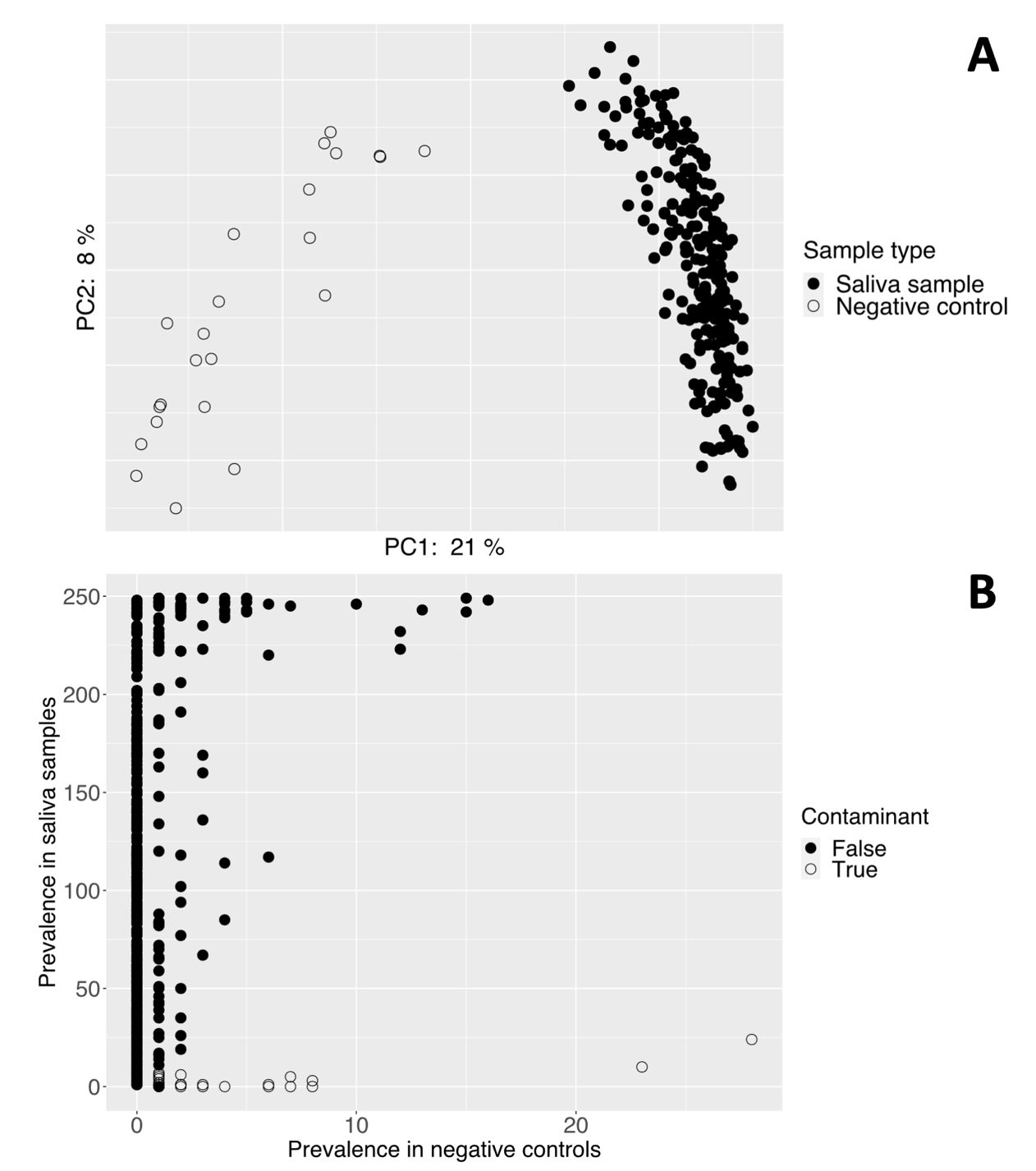
**

**Supplementary Table 1. List of metadata factors uses for microbiota analyses.** All factors listed were initially tested for significant associations with alpha (Faith’s phylogenetic diversity) and beta (unweighted UniFrac) diversity. Factors that returned a statistically significant result are detailed in Table 2 of the main text. In some cases, information content is duplicated across two categories where numerically-coded data have been recoded for ease of interpretation (see, for example, “BrushingPerDay” cf “BrushingPerDay_cat”). These duplicate categories are marked by the addition of “_cat” at the end of the category name.

| **Category name** | **Explanation** | **Possible values** |
| --- | --- | --- |
| Age | Participant’s stated age on questionnaire | 4-17 |
| ExaminationDate | Date of dental examination. Values marked with a ‘*’ contained at least 10 samples and were re-tested separately to minimise the effect of variation in sample size across different dates. | 18-May-15, 9-Aug-15, 11-Aug-15*, 12-Aug-15*, 13-Aug-15*, 14-Aug-15, 17-Aug-15*, 18-Aug-15,* 19-Aug-15*, 20-Aug-15*, 21-Aug-15, 23-Aug-15, 24-Aug-15,* 25-Aug-15*, 26-Aug-15, 27-Aug-15, 28-Aug-15, 10-Sep-15, 12-Sep-15, 17-Sep-15, 22-Sep-15, 1-Oct-15, 12-Oct-15, 13-Oct-15, 19-Oct-15, 21-Oct-15, 22-Oct-15, 28-Oct-15, 12-Nov-15, 18-Nov-15, unknown |
| Examiner | Initials of dental professional who performed dental examination | DG, JR, KK, LC |
| Saliva_Consistency | Based on visual inspection of stimulated saliva sample | 1 (increased: “sticky, frothy saliva residues”), 2 (medium: “frothy, bubbly saliva, increased viscosity”), 3 (normal: “watery, clear saliva, normal viscosity”), - |
| Saliva_pHMMGC | Stimulated saliva pH | continuous |
| Saliva_QuantityInFiveMin_mL | Stimulated saliva flow rate | continuous |
| Saliva_TotalBufferingCapacity | Stimulated saliva buffering capacity | continuous |
| Gender | Participant’s stated gender on questionnaire | 1 (male), 2 (female), unknown |
| CariesStatus | Binary caries status (based on highest ICDAS value recorded for that participant) | CariesFree, CariesActive, NotRecorded |
| NoSurfacesCariesTotal | Total number of tooth surfaces with caries activity recorded (ICDAS score >0) | continuous |
| CariesSeverity | Maximum caries severity observed for participant (based on highest ICDAS value recorded for that participant) | sound, incipient, moderate, severe, NotRecorded |
| GeneralHealth | Response to “Do you think your general health is … ?” | excellent, very_good, good, fair, poor, - |
| OralHealth | Response to “Do you think your dental health is … ?” | excellent, very_good, good, fair, poor, - |
| DentalHealthImportance | Response to “On a scale of 1 to 10, where would you rate the importance of your dental health” | 1 (most important) - 10 (least important), - |
| Toothbrush | Response to “Do you have a toothbrush?” | Y, N, unknown |
| BrushingPerDay | Response to “How often do you brush your teeth?” | 1, 2, 3, 4, - |
| BrushingPerDay_cat | Response to “How often do you brush your teeth?” | less than once a day, once, twice, more than twice a day, unknown |
| BrushingLastWeek | Response to “In the last week, how often did you brush your teeth?” | 1, 2, 3, 4, - |
| BrushingLastWeek_cat | Response to “In the last week, how often did you brush your teeth?” | less than once a day, once, twice, more than twice a day, unknown |
| UseToothpaste | Response to “How often do you use toothpaste when brushing your teeth?” | 1, 2, 3, 4, - |
| UseToothpaste_cat | Response to “How often do you use toothpaste when brushing your teeth?” | always, most times, sometimes, never, unknown |
| PasteBrand | Response to “What brand of toothpaste do you use?” | free response |
| BushMedicine | Response to “Do you use bush medicine/food* to look after your teeth?”  *i.e. traditional medicines | Y, N, unknown |
| DentistUsualReason | Response to “What is the reason why you usually visit the dental clinic?” | Y, N, unknown |
| LastDentalVisit | Response to “When was the last time you visited the dental clinic?” | Y, N, unknown |
| LastDentalVisit_cat | Response to “When was the last time you visited the dental clinic?” | Y, N, unknown |
| ReasonForLastDentalVisit | Response to “The last time you went to the dental clinic, what was the reason?” | Y, N, unknown |
| Diet_Fruit | Response to “On a typical day, do you have any of the following … ?” | Y, N, unknown |
| Diet_Veg | Response to “On a typical day, do you have any of the following … ?” | Y, N, unknown |
| Diet_Meat | Response to “On a typical day, do you have any of the following … ?” | Y, N, unknown |
| Diet_Dairy | Response to “On a typical day, do you have any of the following … ?” | Y, N, unknown |
| Diet_SoftDrink | Response to “On a typical day, do you have any of the following … ?” | Y, N, unknown |
| Diet_Sweets | Response to “On a typical day, do you have any of the following … ?” | Y, N, unknown |
| Diet_SyrupsJamsSpreads | Response to “On a typical day, do you have any of the following … ?” | Y, N, unknown |
| Diet_AddSugarToFoodDrinks | Response to “Do you add sugar to your cereal, tea, coffee or Milo?” | Y, N, unknown |
| Diet_HowManyTspAddedSugar | Response to “How many teaspoons do you add in total?” | continuous |
| Emotion_FeelWorried | Response to General Child Quality of Life statements on questionnaire | 1 (You don’t feel *worried* today), 2 (You feel a little bit *worried* today), 3 (You feel a bit *worried* today), 4 (You feel quite *worried* today), 5 (You feel very *worried* today), unknown |
| Emotion_FeelSad | Response to General Child Quality of Life statements on questionnaire | Refer to Emotion_FeelWorried, substituting *worried* for *sad* |
| Emotion_FeelPain | Response to General Child Quality of Life statements on questionnaire | Refer to Emotion_FeelWorried, substituting *worried* for *pain* |
| Emotion_FeelTired | Response to General Child Quality of Life statements on questionnaire | Refer to Emotion_FeelWorried, substituting *worried* for *tired* |
| EmotionFeelAnnoyed | Response to General Child Quality of Life statements on questionnaire | Refer to Emotion_FeelWorried, substituting *worried* for *annoyed* |
| Emotion_SchoolWorkProblems | Response to General Child Quality of Life statements on questionnaire | 1 (You have no problems with your *schoolwork/homework* today), 2 (You have a few problems with your *schoolwork/homework* today), 3 (You have some problems with your *schoolwork/homework* today), 4 (You have many problems with your *schoolwork/homework* today), 5 (You can’t do your *schoolwork/homework* today), unknown |
| Emotion_Sleep | Response to General Child Quality of Life statements on questionnaire | Refer to Emotion_SchoolWorkProblems, substituting *schoolwork/homework* for *sleeping* |
| Emotion_DailyRoutine | Response to General Child Quality of Life statements on questionnaire | Refer to Emotion_SchoolWorkProblems, substituting *schoolwork/homework* for *your daily routine* |
| Emotion_JoiningActivities | Response to General Child Quality of Life statements on questionnaire | 1 (You can join in with any activities today), 2 (You can join in with most activities today), 3 (You can join in with some activities today), 4 (You can join in with a few activities today), 5 (You can join in with no activities today), unknown |
| OHIP_TroublePronouncing | Response to “Have you had trouble pronouncing words because of problems with your teeth, mouth or dentures?” | 0 (never), 1 (hardly ever), 2 (occasionally), 3 (fairly often), 4 (very often), unknown |
| OHIP_Taste | Response to “Have you felt that your sense of taste has worsened because of problems with your teeth, mouth or dentures?” | 0 (never), 1 (hardly ever), 2 (occasionally), 3 (fairly often), 4 (very often), unknown |
| OHIP_Ache | Response to “Have you had painful aching in your mouth?” | 0 (never), 1 (hardly ever), 2 (occasionally), 3 (fairly often), 4 (very often), unknown |
| OHIP_EatingProblems | Response to “Have you found it uncomfortable to eat any foods because of problems with your teeth, mouth or dentures?” | 0 (never), 1 (hardly ever), 2 (occasionally), 3 (fairly often), 4 (very often), unknown |
| OHIP_SelfConscious | Response to “Have you felt self-conscious because of problems with your teeth, mouth or dentures?” | 0 (never), 1 (hardly ever), 2 (occasionally), 3 (fairly often), 4 (very often), unknown |
| OHIP_Tense | Response to “Have you felt tense because of problems with your teeth, mouth or dentures?” | 0 (never), 1 (hardly ever), 2 (occasionally), 3 (fairly often), 4 (very often), unknown |
| OHIP_DietProblems | Response to “Has your diet been unsatisfactory because of problems with your teeth, mouth or dentures?” | 0 (never), 1 (hardly ever), 2 (occasionally), 3 (fairly often), 4 (very often), unknown |
| OHIP_InterruptMeals | Response to “Have you had to interrupt meals because of problems with your teeth, mouth or dentures?” | 0 (never), 1 (hardly ever), 2 (occasionally), 3 (fairly often), 4 (very often), unknown |
| OHIP_DifficultToRelax | Response to “Have you found it difficult to relax because of problems with your teeth, mouth or dentures?” | 0 (never), 1 (hardly ever), 2 (occasionally), 3 (fairly often), 4 (very often), unknown |
| OHIP_Embarrassed | Response to “Have you been a bit embarrassed because of problems with your teeth, mouth or dentures?” | 0 (never), 1 (hardly ever), 2 (occasionally), 3 (fairly often), 4 (very often), unknown |
| OHIP_Irritable | Response to “Have you been a bit irritable with other people because of problems with your teeth, mouth or dentures?” | 0 (never), 1 (hardly ever), 2 (occasionally), 3 (fairly often), 4 (very often), unknown |
| OHIP_WorkDifficulty | Response to “Have you had difficulty doing your usual jobs because of problems with your teeth, mouth or dentures?” | 0 (never), 1 (hardly ever), 2 (occasionally), 3 (fairly often), 4 (very often), unknown |
| OHIP_LifeLessSatisfying | Response to “Have you felt that life in general was less satisfying because of problems with your teeth, mouth or dentures?” | 0 (never), 1 (hardly ever), 2 (occasionally), 3 (fairly often), 4 (very often), unknown |
| OHIP_UnableToFunction | Response to “Have you been totally unable to function because of problems with your teeth, mouth or dentures?” | 0 (never), 1 (hardly ever), 2 (occasionally), 3 (fairly often), 4 (very often), unknown |
| Housing_HowManyPeople | Response to “How many people do you live with?” | 1 (one to five), 2 (six to ten), 3 (more than 10), - |
| Housing_HowManyPeople_cat | Response to “How many people do you live with?” | 1to5, 6to10, MoreThan10, unknown |
| Housing_HowManyWork | Response to “How many people that you live with go to work?” | none, more_than_one, unknown |

**Supplementary Table 2.** Detailed data for continuous metadata variables from individuals who donated the 205 saliva samples used for microbiota analysis. The number of samples and percentage of the total for each recorded value are reported; percentage values are rounded to one decimal place and may not add to exactly 100%.

| **Category** | **Values** | **n** | **%** |
| --- | --- | --- | --- |
| Age |  |  |  |
|  | 4 | 20 | 9.8% |
|  | 5 | 22 | 10.7% |
|  | 6 | 25 | 12.2% |
|  | 7 | 32 | 15.6% |
|  | 8 | 21 | 10.2% |
|  | 9 | 17 | 8.3% |
|  | 10 | 16 | 7.8% |
|  | 11 | 11 | 5.4% |
|  | 12 | 5 | 2.4% |
|  | 13 | 9 | 4.4% |
|  | 14 | 4 | 2.0% |
|  | 15 | 10 | 4.9% |
|  | 16 | 5 | 2.4% |
|  | 17 | 6 | 2.9% |
|  | Unknown or not recorded | 2 | 1.0% |
| Saliva pH |  |  |  |
|  | 5.4 | 1 | 0.5% |
|  | 5.6 | 3 | 1.5% |
|  | 6 | 2 | 1.0% |
|  | 6.1 | 1 | 0.5% |
|  | 6.2 | 4 | 2.0% |
|  | 6.4 | 20 | 9.8% |
|  | 6.6 | 2 | 1.0% |
|  | 6.8 | 40 | 19.5% |
|  | 7 | 26 | 12.7% |
|  | 7.2 | 19 | 9.3% |
|  | 7.4 | 26 | 12.7% |
|  | 7.6 | 37 | 18.0% |
|  | 7.7 | 1 | 0.5% |
|  | 7.8 | 6 | 2.9% |
|  | Unknown or not recorded | 17 | 8.3% |
| Saliva flow rate (mL per 5 minutes) |  |  |  |
|  | 0.5 | 1 | 0.5% |
|  | 1 | 5 | 2.4% |
|  | 1.5 | 2 | 1.0% |
|  | 2 | 13 | 6.3% |
|  | 2.5 | 3 | 1.5% |
|  | 3 | 11 | 5.4% |
|  | 3.5 | 22 | 10.7% |
|  | 4 | 11 | 5.4% |
|  | 4.5 | 3 | 1.5% |
|  | 5 | 32 | 15.6% |
|  | 5.5 | 2 | 1.0% |
|  | 6 | 22 | 10.7% |
|  | 7 | 23 | 11.2% |
|  | 8 | 12 | 5.9% |
|  | 8.5 | 1 | 0.5% |
|  | 9 | 8 | 3.9% |
|  | 9.5 | 1 | 0.5% |
|  | 10 | 5 | 2.4% |
|  | 11 | 6 | 2.9% |
|  | 11.5 | 1 | 0.5% |
|  | 12 | 3 | 1.5% |
|  | 14 | 3 | 1.5% |
|  | 15 | 2 | 1.0% |
|  | 16 | 1 | 0.5% |
|  | Unknown or not recorded | 11 | 5.4% |
| Total carious surfaces |  |  |  |
|  | 0 | 17 | 8.3% |
|  | 1 | 14 | 6.8% |
|  | 2 | 14 | 6.8% |
|  | 3 | 16 | 7.8% |
|  | 4 | 10 | 4.9% |
|  | 5 | 7 | 3.4% |
|  | 6 | 11 | 5.4% |
|  | 7 | 8 | 3.9% |
|  | 8 | 16 | 7.8% |
|  | 9 | 9 | 4.4% |
|  | 10 | 10 | 4.9% |
|  | 11 | 13 | 6.3% |
|  | 12 | 5 | 2.4% |
|  | 13 | 4 | 2.0% |
|  | 14 | 5 | 2.4% |
|  | 15 | 5 | 2.4% |
|  | 16 | 5 | 2.4% |
|  | 17 | 4 | 2.0% |
|  | 18 | 3 | 1.5% |
|  | 19 | 3 | 1.5% |
|  | 20 | 1 | 0.5% |
|  | 21 | 2 | 1.0% |
|  | 22 | 2 | 1.0% |
|  | 24 | 4 | 2.0% |
|  | 25 | 1 | 0.5% |
|  | 26 | 1 | 0.5% |
|  | 27 | 1 | 0.5% |
|  | 29 | 1 | 0.5% |
|  | 33 | 1 | 0.5% |
|  | 37 | 3 | 1.5% |
|  | 39 | 2 | 1.0% |
|  | 42 | 1 | 0.5% |
|  | 56 | 1 | 0.5% |
|  | 62 | 1 | 0.5% |
|  | Unknown or not recorded | 4 | 2.0% |
|  |  |  |  |
| TOTAL |  | 205 | 100% |

**Supplementary Table 3. List of microbial features statistically identified as contaminants using *decontam*.** The feature ID and feature taxonomy as assigned using the SILVA 132 database are displayed. Features were identified as contaminants in *decontam* if they were more prevalent in negative controls (EBCs and NTCs) than in saliva samples.

| **Feature ID** | **Feature taxonomy** |
| --- | --- |
| 592d5efeb0a3464332b2cfc17efd4c2a | D_0__Bacteria;D_1__Proteobacteria;D_2__Gammaproteobacteria;D_3__Xanthomonadales;D_4__Xanthomonadaceae;D_5__Stenotrophomonas |
| ca7004f0828295e2d362a3d91c8eddbf | D_0__Bacteria;D_1__Proteobacteria;D_2__Gammaproteobacteria;D_3__Betaproteobacteriales;D_4__Burkholderiaceae;D_5__Comamonas |
| 7a36f3ebcbcb5d51d4b69105fc60093f | D_0__Bacteria;D_1__Proteobacteria;D_2__Gammaproteobacteria;D_3__Betaproteobacteriales;D_4__Burkholderiaceae |
| 4efd6871877830573a55339de7cfd3dd | D_0__Bacteria;D_1__Proteobacteria;D_2__Gammaproteobacteria;D_3__Betaproteobacteriales;D_4__Neisseriaceae;D_5__Neisseria;D_6__uncultured bacterium |
| f63e7fbddd1944b3a5adf3bbbccd71e8 | D_0__Bacteria;D_1__Proteobacteria;D_2__Gammaproteobacteria;D_3__Betaproteobacteriales;D_4__Burkholderiaceae;D_5__Burkholderia-Caballeronia-Paraburkholderia |
| c1dedbc2ee20ed310022b9a1976d56c4 | D_0__Bacteria;D_1__Proteobacteria;D_2__Gammaproteobacteria;D_3__Betaproteobacteriales;D_4__Burkholderiaceae;D_5__Pelomonas;D_6__uncultured bacterium |
| 82dab61dd63598195bc7cb978ec6c0e5 | D_0__Bacteria;D_1__Proteobacteria;D_2__Gammaproteobacteria;D_3__Betaproteobacteriales;D_4__Burkholderiaceae;D_5__Noviherbaspirillum;D_6__uncultured bacterium |
| 007ac2c602df5b6ad5d7a3293b682254 | D_0__Bacteria;D_1__Proteobacteria;D_2__Gammaproteobacteria;D_3__Betaproteobacteriales;D_4__Burkholderiaceae;D_5__Tepidimonas;D_6__uncultured bacterium |
| 62bf37c50994a06e6c8c93355ad1caa0 | D_0__Bacteria;D_1__Proteobacteria;D_2__Gammaproteobacteria;D_3__Pseudomonadales;D_4__Pseudomonadaceae;D_5__Pseudomonas |
| 678e401ab6a2b69c430051f97d6de792 | D_0__Bacteria;D_1__Proteobacteria;D_2__Gammaproteobacteria;D_3__Pseudomonadales;D_4__Pseudomonadaceae;D_5__Pseudomonas |
| 5747992ec8974831558f048e9924dad4 | D_0__Bacteria;D_1__Proteobacteria;D_2__Gammaproteobacteria;D_3__Pseudomonadales;D_4__Pseudomonadaceae;D_5__Pseudomonas |
| bc216d958df9f369feac8c3f52bd9a68 | D_0__Bacteria;D_1__Proteobacteria;D_2__Gammaproteobacteria;D_3__Pseudomonadales;D_4__Pseudomonadaceae;D_5__Pseudomonas |
| 4ef42e59c16ac771bbf589e63cf3f3fa | D_0__Bacteria;D_1__Proteobacteria;D_2__Gammaproteobacteria;D_3__Alteromonadales;D_4__Shewanellaceae;D_5__Shewanella;D_6__Shewanella algae |
| 85e71841509f6b3c0956f537cf3b3220 | D_0__Bacteria;D_1__Proteobacteria;D_2__Gammaproteobacteria;D_3__Pasteurellales;D_4__Pasteurellaceae;D_5__Haemophilus;D_6__uncultured bacterium |
| 95eda7cc1f12a89d5bd3e2439fec64f3 | D_0__Bacteria;D_1__Proteobacteria;D_2__Gammaproteobacteria;D_3__Pseudomonadales;D_4__Moraxellaceae;D_5__Acinetobacter;D_6__Acinetobacter baumannii |
| 44cc0863838fe6b620b8a06323f46c94 | D_0__Bacteria;D_1__Proteobacteria;D_2__Alphaproteobacteria;D_3__Sphingomonadales;D_4__Sphingomonadaceae;D_5__Erythrobacter;D_6__uncultured bacterium |
| efada544d18b18374c77aa7c65bc4fd0 | D_0__Bacteria;D_1__Proteobacteria;D_2__Alphaproteobacteria;D_3__Sphingomonadales;D_4__Sphingomonadaceae;D_5__Novosphingobium |
| 94f0270bf61e49c97e7fb34688750dce | D_0__Bacteria;D_1__Proteobacteria;D_2__Alphaproteobacteria;D_3__Sphingomonadales;D_4__Sphingomonadaceae;D_5__Sphingomonas |
| a6ace26d1c29f5de5f29bc02990902d8 | D_0__Bacteria;D_1__Proteobacteria;D_2__Alphaproteobacteria;D_3__Sphingomonadales;D_4__Sphingomonadaceae;D_5__Polymorphobacter;D_6__uncultured bacterium |
| 7f4909ae8d31a3995fa2c200c690a21f | D_0__Bacteria;D_1__Proteobacteria;D_2__Alphaproteobacteria;D_3__Caulobacterales;D_4__Caulobacteraceae;D_5__Brevundimonas |
| cfe7782ee55e41fbc4e805e67f44422c | D_0__Bacteria;D_1__Proteobacteria;D_2__Alphaproteobacteria;D_3__Rhizobiales;D_4__Rhizobiaceae;D_5__Mesorhizobium |
| 731fad9f7b7afa6616ae0ee51b19afec | D_0__Bacteria;D_1__Proteobacteria;D_2__Alphaproteobacteria;D_3__Rhizobiales;D_4__Xanthobacteraceae;D_5__Bradyrhizobium |
| b18b389447d758e7cb173b4b4f2ad960 | D_0__Bacteria;D_1__Proteobacteria;D_2__Alphaproteobacteria;D_3__Rhizobiales;D_4__Xanthobacteraceae;D_5__Bradyrhizobium |
| 2aad4b5449d0a7c0291f2ce80ae2db1f | D_0__Bacteria;D_1__Proteobacteria;D_2__Alphaproteobacteria;D_3__Rhizobiales;D_4__Rhizobiales Incertae Sedis;D_5__Phreatobacter;D_6__uncultured bacterium |
| 2ca928ad9749bb9726c35d6528fefec1 | D_0__Bacteria;D_1__Proteobacteria;D_2__Alphaproteobacteria;D_3__Rhizobiales;D_4__Beijerinckiaceae;D_5__Methylobacterium;D_6__uncultured bacterium |
| 729adc884c6f1c451cb3e125d69084f2 | D_0__Bacteria;D_1__Proteobacteria;D_2__Alphaproteobacteria;D_3__Rhizobiales;D_4__Beijerinckiaceae;D_5__Bosea;D_6__uncultured bacterium |
| aa25a8c74fea2a46ad2df4977d04a45e | D_0__Bacteria;D_1__Bacteroidetes;D_2__Bacteroidia;D_3__Bacteroidales;D_4__Prevotellaceae;D_5__Prevotella 7;D_6__uncultured bacterium |
| 3ff456f2bde1f0d155ce3d80180aa757 | D_0__Bacteria;D_1__Bacteroidetes;D_2__Bacteroidia;D_3__Sphingobacteriales;D_4__env.OPS 17;D_5__uncultured eubacterium env.OPS 17;D_6__uncultured eubacterium env.OPS 17 |
| f8753b9f13abb306b57aedce2cf6e7d0 | D_0__Bacteria;D_1__Bacteroidetes;D_2__Bacteroidia;D_3__Flavobacteriales;D_4__Weeksellaceae;D_5__Cloacibacterium;D_6__uncultured bacterium |
| 2cfba508362460cee3bb13837abd48b1 | D_0__Bacteria;D_1__Actinobacteria;D_2__Actinobacteria;D_3__Corynebacteriales;D_4__Corynebacteriaceae;D_5__Corynebacterium 1 |
| 4c4606a3dbb4f5b8bb1ad1db6dfc5617 | D_0__Bacteria;D_1__Actinobacteria;D_2__Actinobacteria;D_3__Propionibacteriales;D_4__Propionibacteriaceae;D_5__Cutibacterium |
| cb2fe0146e2fbcb101050edb996a0ee2 | D_0__Bacteria;D_1__Firmicutes;D_2__Bacilli;D_3__Bacillales;D_4__Staphylococcaceae;D_5__Staphylococcus |
| 393f255ee6bc09b823094bd9b4e818a8 | D_0__Bacteria;D_1__Firmicutes;D_2__Bacilli;D_3__Bacillales;D_4__Paenibacillaceae;D_5__Paenibacillus |
| cebe523e0c1eb417307ebe6108ded444 | D_0__Bacteria;D_1__Firmicutes;D_2__Bacilli;D_3__Lactobacillales;D_4__Lactobacillaceae;D_5__Lactobacillus |
| d470c1038d8fe0ca897b03f327320ace | D_0__Bacteria;D_1__Firmicutes;D_2__Bacilli;D_3__Lactobacillales;D_4__Carnobacteriaceae;D_5__Atopostipes;D_6__uncultured bacterium |
| dab4d3e6078cfc510502a47c98e82081 | D_0__Bacteria;D_1__Firmicutes;D_2__Negativicutes;D_3__Selenomonadales;D_4__Veillonellaceae;D_5__Veillonella |
| 38f72169844b5d2ee8dbd40fa4a5bd36 | D_0__Bacteria;D_1__Cyanobacteria;D_2__Oxyphotobacteria;D_3__Nostocales;D_4__Coleofasciculaceae;D_5__uncultured;D_6__uncultured bacterium |
| 5e69019c3b54d023d3bdf70ad04f6d88 | D_0__Bacteria;D_1__Cyanobacteria;D_2__Oxyphotobacteria;D_3__Oxyphotobacteria Incertae Sedis;D_4__Unknown Family;D_5__Phormidium SAG 37.90;D_6__uncultured bacterium |
| 8145e7cc4dc46344a9c7b4139eaefb3f | D_0__Bacteria;D_1__Cyanobacteria;D_2__Oxyphotobacteria;D_3__Oxyphotobacteria Incertae Sedis;D_4__Unknown Family;D_5__Oscillatoria SAG 1459-8;D_6__uncultured bacterium |

**Supplementary Table 4. Comparison of taxonomic composition of salivary microbiota using SILVA and HOMD databases**. Summaries of the overall taxonomic composition of salivary microbiota are listed at the phylum and genus levels, comparing taxonomic classifications obtained using the SILVA 132 and Human Oral Microbiome Database (HOMD) v15.1 databases. Results that differ between the two databases are marked with an asterisk (*). Percentage values are rounded to the nearest whole number and may not sum to 100%.

| **Phylum level** | | | |
| --- | --- | --- | --- |
| ***SILVA 132 database*** | | ***HOMD v15.1 database*** | |
| Proteobacteria | 30%* | Proteobacteria | 31%* |
| Bacteroidetes | 26% | Bacteroidetes | 26% |
| Firmicutes | 25% | Firmicutes | 25% |
| Actinobacteria | 12% | Actinobacteria | 12% |
| Fusobacteria | 6% | Fusobacteria | 6% |
| Epsilonbacteraeoata*, Spirochaetes, Patescibacteria*, Tenericutes*, Synergistetes, Cyanobacteria*, Chloroflexi, unassigned Bacteria | remaining 1% * | Spirochaetes, Absconditabacteria (SR1)*, unassigned Bacteria, Synergistetes, Saccharibacteria (TM7)*, Gracilibacteria (GN02)*, Chloroflexi | remaining <1% * |
| **Genus level** | | | |
| ***SILVA 132 database*** | | ***HOMD v15.1 database*** | |
| Prevotella | 18% * | Prevotella | 19% * |
| Neisseria | 14% | Neisseria | 14% |
| Haemophilus | 12% | Haemophilus | 12% |
| Streptococcus | 9% | Streptococcus | 9% |
| Rothia | 8% | Rothia | 8% |
| Veillonella | 6% | Veillonella | 6% |
| Fusobacterium | 4% | Fusobacterium | 4% |
| Alloprevotella | 3% | Alloprevotella | 3% |
| Porphyromonas | 3% | Porphyromonas | 3% |
| Gemella | 2% | Gemella | 2% |
| Granulicatella | 2% | Granulicatella | 2% |
| Leptotrichia | 2% | Leptotrichia | 2% |
| Actinomyces | 2% | Actinomyces | 2% |
| Aggregatibacter | 2% | Aggregatibacter | 2% |
| other genera accounting for <2% of total sequences | remaining 13% * | other genera accounting for <2% of total sequences | remaining 12% * |

**Supplementary Figure 2**. Alpha diversity (Faith’s phylogenetic diversity) plots for significant factors not included in Fig. 2 (salivary pH, salivary flow rate, total carious surfaces, examination date), with results of Spearman correlation (continuous) and Kruskal-Wallis (categorical) tests. For categorical variables (examination date), only values with a minimum of 10 samples were retained for significance testing.

**
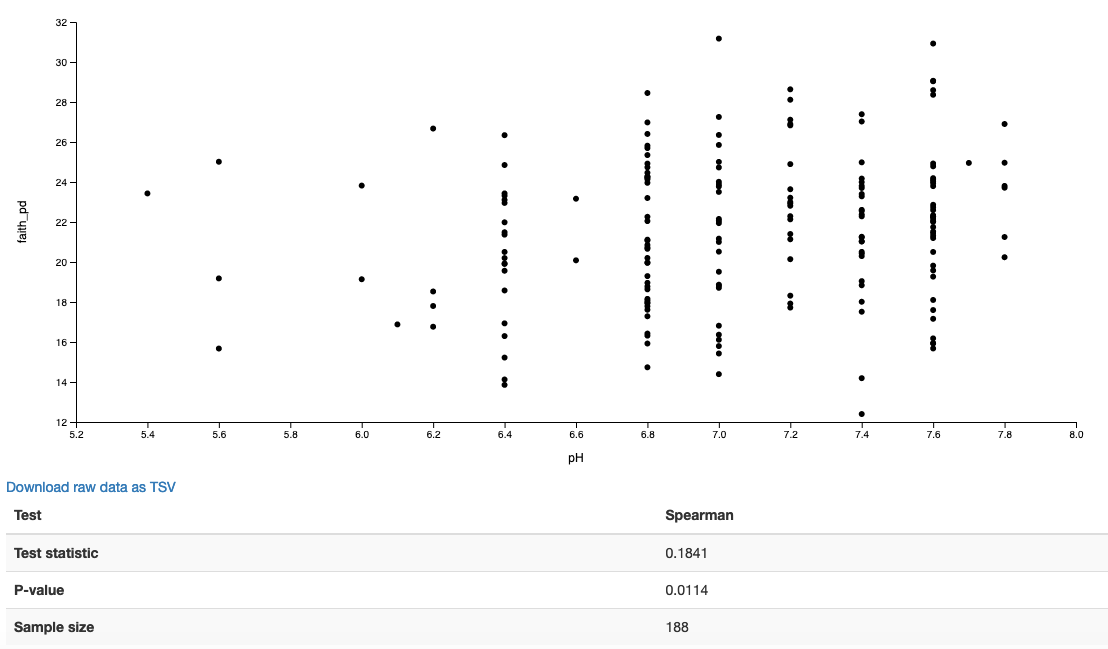
**

**
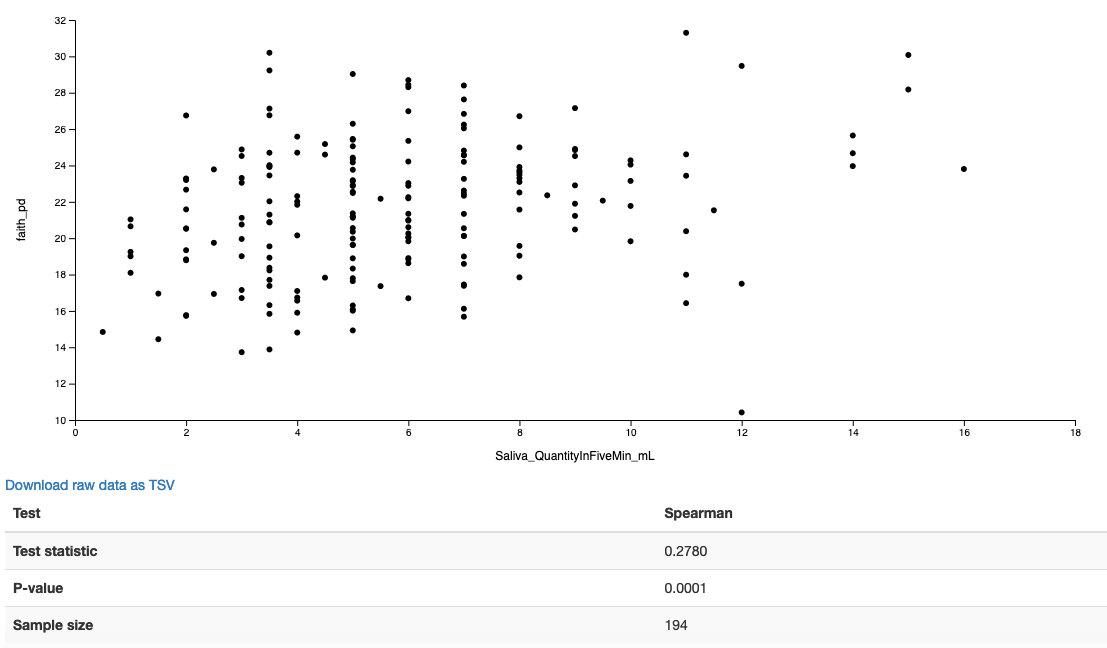

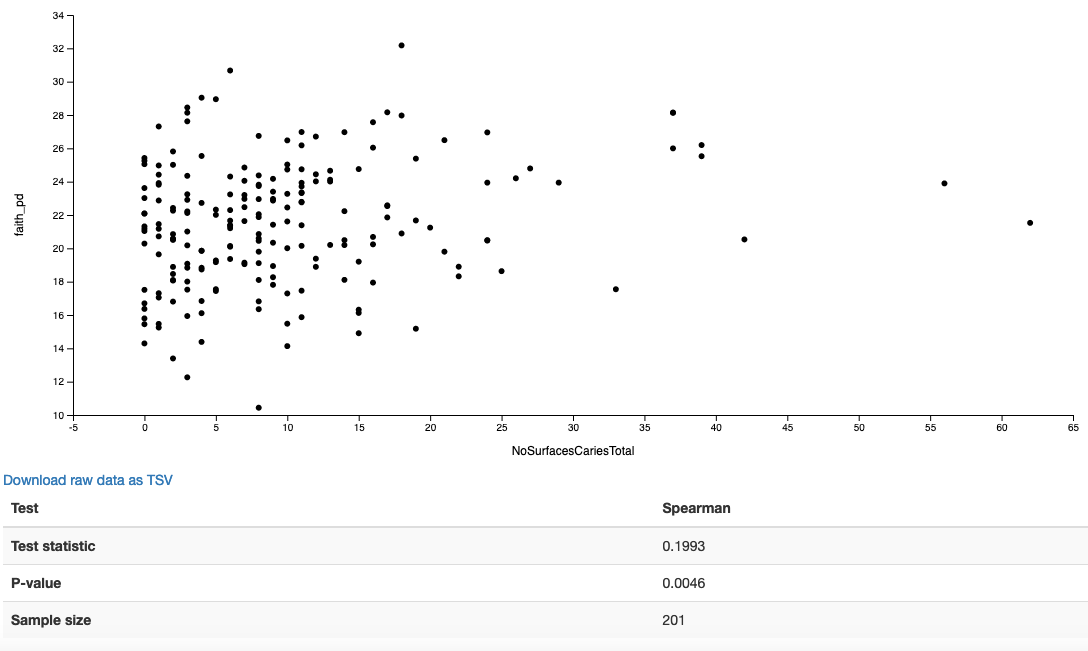

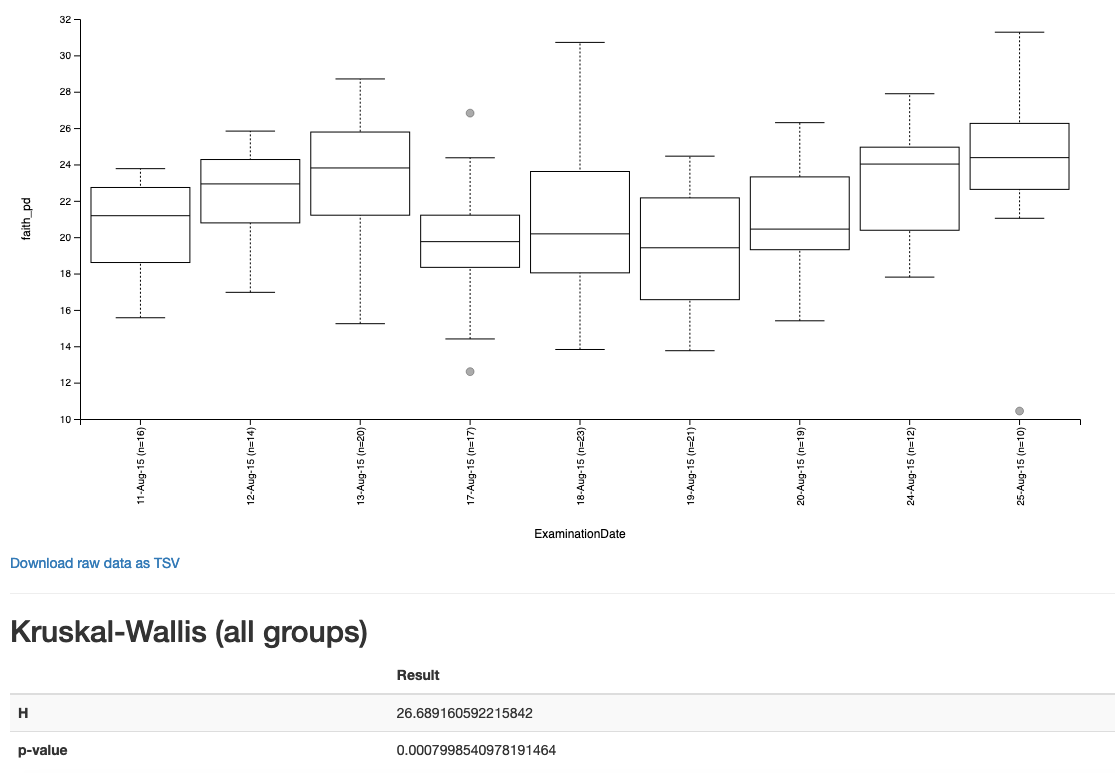
**

**Supplementary Table 5. Detailed list of microbial features differing significantly in abundance across groups identified by ANCOM.** Specific microbial features significantly associated with daily toothbrushing behaviour, caries status, caries severity, and examination date are listed below. The metadata category, feature ID, full-length feature taxonomy string as assigned using the SILVA 132 database, the reported W-value from ANCOM testing, and the overall prevalence (*i.e.* number of samples the feature was detected in) and abundance (*i.e.* total number of sequences associated with the feature) in the dataset are displayed for each significant feature. Samples with unknown or unrecorded values for a given category were removed prior to ANCOM testing.

| **Category** | **Feature ID** | **Taxonomy** | **ANCOM W-value** | **Prevalence** | **Abundance** |
| --- | --- | --- | --- | --- | --- |
| Daily toothbrushing | 5122bc491a9dda8710f98d0077187095 | Bacteria; Firmicutes; Bacilli; Lactobacillales; Streptococcaceae; Streptococcus; Streptococcus sobrinus | 279 | 51 | 433 |
| Caries status | a14e8dc28187e7dc1d39dae36dec8dc6 | Bacteria; Proteobacteria; Gammaproteobacteria; Pasteurellales; Pasteurellaceae; Actinobacillus; Actinobacillus porcinus | 81 | 5 | 8649 |
|  | 1f550fa73b5832b9e4144289c64332d4 | Bacteria; Fusobacteria; Fusobacteriia; Fusobacteriales; Leptotrichiaceae; Leptotrichia | 26 | 11 | 121 |
|  | 047b7fb62a5e9d2711e639ae1cb1519a | Bacteria; Firmicutes; Negativicutes; Selenomonadales; Veillonellaceae; Veillonella; uncultured organism | 19 | 195 | 24,662 |
| Caries severity | 7c57afe3b6b21af9d22cea9e92af463b | Bacteria; Firmicutes; Lactobacillales; Lactobacillaceae; Lactobacillus; Lactobacillus gasseri | 208 | 44 | 1,660 |
| Examination date | 2e6693c82c27b86e6c2e720ec1d7c95f | Bacteria; Actinobacteria; Actinobacteria; Actinomycetales; Actinomycetaceae; Actinomyces; uncultured bacterium | 622 | 59 | 3,978 |

**Supplementary Table 6. Comparison of taxonomic classification of significantly differentially abundant microbial features using SILVA and HOMD databases.** Taxonomic classifications of microbial features discussed in the main text identified as significantly differentially abundant across metadata categories using ANCOM, comparing taxonomic classifications obtained using the SILVA 132 and Human Oral Microbiome Database (HOMD) v15.1 databases. Classifications are given at the most precise taxonomic level permitted by the relevant database. Classifications that differ between databases at the genus level or above are marked with an asterisk (*).

| **Feature ID** | **Significantly associated metadata category** | **SILVA 132 classification** | **HOMD v15.1 classification** |
| --- | --- | --- | --- |
| 2e6693c82c27b86e6c2e720ec1d7c95f | Examination date | D_0__Bacteria;  D_1__Actinobacteria  D_2__Actinobacteria;  D_3__Actinomycetales;  D_4__Actinomycetaceae;  D_5__Actinomyces;  D_6__uncultured bacterium | k__Bacteria;  p__Actinobacteria;  c__Actinobacteria;  o__Actinomycetales;  f__Actinomycetaceae;  g__Actinomyces |
| 5122bc491a9dda8710f98d0077187095 | Daily toothbrushing behavior | D_0__Bacteria;  D_1__Firmicutes;  D_2__Bacilli;  D_3__Lactobacillales;  D_4__Streptococcaceae; D_5__Streptococcus; D_6__Streptococcus sobrinus | k__Bacteria;  p__Firmicutes;  c__Bacilli;  o__Lactobacillales; f__Streptococcaceae; g__Streptococcus |
| a14e8dc28187e7dc1d39dae36dec8dc6 | Caries status | D_0__Bacteria;  D_1__Proteobacteria; D_2__Gammaproteobacteria; D_3__Pasteurellales; D_4__Pasteurellaceae; D_5__Actinobacillus;  D_6__Actinobacillus porcinus * | k__Bacteria;  p__Proteobacteria; c__Gammaproteobacteria; o__Pasteurellales;  f__Pasteurellaceae;  g__Haemophilus * |
| 1f550fa73b5832b9e4144289c64332d4 | Caries status | D_0__Bacteria;  D_1__Fusobacteria; D_2__Fusobacteriia; D_3__Fusobacteriales; D_4__Leptotrichiaceae; D_5__Leptotrichia | k__Bacteria;  p__Fusobacteria;  c__Fusobacteriia;  o__Fusobacteriales; f__Leptotrichiaceae;  g__Leptotrichia;  s__sp._HMT_221 |
| 047b7fb62a5e9d2711e639ae1cb1519a | Caries status | D_0__Bacteria;  D_1__Firmicutes; D_2__Negativicutes; D_3__Selenomonadales; D_4__Veillonellaceae; D_5__Veillonella;  D_6__uncultured organism | k__Bacteria;  p__Firmicutes;  c__Negativicutes;  o__Veillonellales;  f__Veillonellaceae;  g__Veillonella |
| 7c57afe3b6b21af9d22cea9e92af463b | Caries severity | D_0__Bacteria;  D_1__Firmicutes;  D_2__Bacilli;  D_3__Lactobacillales; D_4__Lactobacillaceae; D_5__Lactobacillus; D_6__Lactobacillus gasseri | k__Bacteria;  p__Firmicutes;  c__Bacilli;  o__Lactobacillales; f__Lactobacillaceae; g__Lactobacillus;  s__gasseri |
